# Bleeding disorders, longer operative time, and nongeneral anesthesia increase are associated with overnight admission after hip arthroscopy

**DOI:** 10.1093/jhps/hnae038

**Published:** 2024-12-25

**Authors:** Jack Zhong, Connor R Crutchfield, Nathan J Lee, John Mueller, Christopher Ahmad, David Trofa, Thomas Sean Lynch

**Affiliations:** Department of Orthopaedic Surgery, Columbia University Irving Medical Center, 622 W. 168th Street - PH 11, New York, NY 10032, United States; Department of Orthopaedic Surgery, New York University Langone Health, 301 E. 17th Street, New York, NY 10010, United States; Department of Orthopaedic Surgery, Columbia University Irving Medical Center, 622 W. 168th Street - PH 11, New York, NY 10032, United States; Department of Orthopaedic Surgery, Sidney Kimmel Medical College at Thomas Jefferson University, 925 Chestnut Street, Philadelphia, PA 19107, United States; Department of Orthopaedic Surgery, Columbia University Irving Medical Center, 622 W. 168th Street - PH 11, New York, NY 10032, United States; Department of Orthopaedic Surgery, Columbia University Irving Medical Center, 622 W. 168th Street - PH 11, New York, NY 10032, United States; Department of Orthopaedic Surgery, Columbia University Irving Medical Center, 622 W. 168th Street - PH 11, New York, NY 10032, United States; Department of Orthopaedic Surgery, Columbia University Irving Medical Center, 622 W. 168th Street - PH 11, New York, NY 10032, United States; Department of Orthopaedic Surgery, Columbia University Irving Medical Center, 622 W. 168th Street - PH 11, New York, NY 10032, United States; Department of Orthopedic Surgery, Henry Ford Health, 2799 W. Grand Blvd, Detroit, MI 48202, United States

## Abstract

Overnight admission is a rare but major complication after hip arthroscopy (HA), and the paucity of data surrounding its causes limits patient education and quality of care. The purpose of this study was to identify risk factors for an unanticipated overnight admission after HA and assess for associated complications. This analysis queried the American College of Surgeons National Surgical Quality Improvement Program database using Current Procedural Terminology codes to identify hip arthroscopies from 2005 to 2017. Patient demographics, perioperative variables, and comorbidities were compared between ambulatory and nonambulatory patients [length of stay (LOS) ≥ 1] using bivariate analysis. Multivariate stepwise logistic regression then identified independent risk factors of adverse outcomes. Linear regression analyzed correlation of LOS with age, operative time, modified fragility index (mFI-5), and year of operation. A total of 2420 cases were included in this study with 400 (16.5%) overnight admissions. The mean subject age was 40 ± 13.9 years old (58.1% female). Admitted patients generally had higher American Society of Anesthesiologists (ASA) scores and a higher mFI-5 index. Multivariate logistic regression showed that mFI-5 > 0, bleeding disorders, operative time >1.5 h, and nongeneral anesthesia were independent risk factors for prolonged hospital stay. Patients aged 31–40 years had decreased risk of LOS ≥1. Nonambulatory surgery was associated with significantly increased risk for any complication, readmission, wound complication, and venous thromboembolism. This analysis demonstrates that operations >1.5 h and increased medical comorbidities predispose patients to greater risk of being admitted to the hospital after HA. Surgeons should consider these data to optimize controllable factors and patient selection to reduce the risk of postoperative admission.

## Introduction

Hip arthroscopy (HA) is a minimally invasive procedure with few major complications, and many patients are discharged from the hospital on the day of surgery [1]. In a study of 6395 patients, however, Malviya *et al*. reported 51.7% were discharged on postop-day 1 after HA [2]. A hospital admission after surgery can be costly and associated with potentially avoidable inpatient complications, such as a hospital-acquired infection or deep vein thrombosis [3, 4]. In fact, the admitted patients commonly developed wound-related complications and pain [2]. Among other factors, while a length of stay (LOS) >4 days has been identified as a risk factor for unplanned readmissions after total knee arthroplasty [5], the same association between longer LOS after HA and early postoperative complications or readmissions has not been established. Notably, the only other study evaluating admission after HA by Du *et al*. did not identify longer LOS as a risk factor for unplanned readmissions in their analysis of 1931 cases [6]. In fact, the authors did not use LOS as a variable in their analysis and captured all admissions within 30 days of the index procedure without differentiating between direct admissions and readmissions [6]. Therefore, it is important to identify the risk factors for hospital admission directly after HA and whether those admissions are associated with higher rates of early complications.

The literature investigating LOS for HA and associated complications is limited. Kay *et al*. stated in a review that when a perioperative femoral nerve block was used rather than opioids for pain control after HA, patients had a significantly decreased LOS, while four other studies showed an increased recovery time when a nerve block was used compared to standard analgesia [7]. Ultimately, the nerve blocks showed mixed effects on LOS after HA, but little focus was given to the risk factors for admission after HA [7]. In a retrospective analysis of 273 hip arthroplasty patients by Sibia *et al*., old age, high BMI, female sex, American Society of Anesthesiologists (ASA) scores >3, and an inability to ambulate on the same day of surgery were associated with increased LOS [3]. Furthermore, Ricci *et al*. reported increased ASA, preoperative cardiac testing, male gender, and admission date of Thursday or Friday were independent predictors of a longer LOS after hip fracture [8]. Although the latter two studies demonstrate certain risk factors for longer LOS after hip surgery, they did not study HA patients or offer a comparison of complication rates between patients with longer vs. shorter LOS [3, 8].

The purpose of this study was to determine risk factors for an unanticipated overnight admission after HA using a comparison of ambulatory vs. nonambulatory patients and assess for any associations with postoperative complications. These data will help surgeons and hospitals to appropriately triage patients preoperatively to avoid unnecessary hospital admissions, reduce costs, and prevent adverse surgical outcomes after HA. The authors hypothesized that a BMI of >30 and medical comorbidities will be risk factors for nonambulatory HA and that patients who stay overnight will have an association with increased postoperative complications.

## Materials and methods

All data were collected from the American College of Surgeons National Surgical Quality Improvement Program (ACS-NSQIP) database, a national, validated database of de-identified patient data collected from its 708 participating hospitals [9]. Data from 2005 to 2017 were used for this study. The database collects a comprehensive set of demographics, medical comorbidities, and perioperative factors up to 30 days after the index procedure, tracking complications such as wound issues, sepsis, venous thromboembolism (VTE), urinary tract infection (UTI), and transfusions [9]. The time range of 2005–17 was used because many of the variables of interest were either not collected after 2017 or their collection was interrupted by the COVID-19 pandemic.

Patient cases included for analysis were queried using the Current Procedural Terminology (CPT) codes defined in Table 1. Patients with CPT codes of 29999 or “Unlisted procedure, arthroscopy” were further identified using the International Classification of Diseases, Ninth Revision (ICD-9) codes as has been done in other HA studies [10]. Nonelective hip arthroscopies, revision cases, and one outlier patient with an LOS of 365 days were excluded from analysis.

**Table 1.** Definitions of CPT and ICD-9 codes queried.

Code	Definition
CPT	
29860	HA, diagnostic with or without biopsy
29861	Arthroscopy, hip, and surgical with removal of loose body or foreign body
29862	HA, chondroplasty, abrasion arthroplasty, and/or resection of labrum
29863	HA, synovectomy, or cleaning out inflammation
29914	HA, femoroplasty and shaving femoral head/neck junction
29915	Pincer lesion performed with acetabuloplasty
29916	HA and labral repair
29999	unlisted arthroscopic procedure
ICD-9	
718.95	Joint derangement, unspecified, pelvic region, and thigh
715.15	Osteoarthrosis, localized, primary, pelvic region, and thigh
715.35	Osteoarthrosis, localized, not specified whether primary or secondary, pelvic region, and thigh
715.95	Osteoarthrosis, unspecified whether generalized or localized, pelvic region, and thigh
716.95	Arthropathy, unspecified, pelvic region, and thigh
718.05	Articular cartilage disorder, pelvic region, and thigh
718.35	Recurrent dislocation of joint, pelvic region, and thigh
718.65	Unspecified intrapelvic protrusion of acetabulum, pelvic region, and thigh
718.85, 719.65, 719.85, and 719.95	Other symptoms, joint derangement, or specified disorders referable to joint, pelvic region, and thigh
719.45	Pain in joint, pelvic region, and thigh
726.5	Enthesopathy of hip region
733.42	Aseptic necrosis of head and neck of femur
736.39	Other specified acquired deformities of unspecified thigh
754.32	Congenital subluxation of hip, unilateral
755.63	Other congenital deformity of hip (joint)
843 and 843.8	Sprains and strains of hip and thigh

Patients were divided into two cohorts based on LOS: nonambulatory or admitted (NA) if LOS ≥1 days and ambulatory (A) if LOS <1 day. Therefore, those discharged on the day of surgery or before postoperative day one were in Group A, while patients staying at least 1 day in the hospital were in Group NA. The two cohorts were compared against each other using patient demographics, medical comorbidities, and perioperative variables. Surgery was either done at an ambulatory surgery center or “outpatient” vs. a hospital or “inpatient” setting. Demographic variables included age (divided into five groups), gender, race, clinical obesity (BMI > 30), ASA score, diabetic status, history of chronic obstructive pulmonary disease (COPD), smoking status within a year of surgery, dyspnea, cardiac comorbidity, renal comorbidity, functional health status, weight loss >10%, hypertension, and steroid use for chronic conditions as outlined in Table 2. The new five-factor modified fragility index (mFI-5) was calculated for both cohorts according to the method described in Sneha *et al*. [11]. Native Hawaiians, Alaskan Natives, and unknown races were combined into the “Other” category.

**Table 2.** Rate of prolonged hospital stay after HA for demographic variables, comorbidities, and intraoperative factors.

	Ambulatory: LOS < 1 day (*N* = 2020)	Nonambulatory: LOS ≥ 1 days (*N* = 400)	Total (*N* = 2420)	*P*-value
Average LOS (SD)	0.0 (0.0)	2.1 (3.2)	0.4 (1.5)	–
Average age: mean (SD)	39.6 (13.3)	41.8 (16.6)	40.0 (13.9)	**.146** ^a^
Age group (years)				**.006** ^b^
18–20	106 (5.2%)	25 (6.3%)	131 (5.4%)	
21–30	458 (22.7%)	99 (24.8%)	557 (23.0%)	
31–40	558 (27.6%)	84 (21.0%)	642 (26.5%)	
41–50	487 (24.1%)	85 (21.3%)	572 (23.6%)	
>50	411 (20.3%)	107 (26.8%)	518 (21.4%)	
Female gender	1174 (58.1%)	231 (57.8%)	1405 (58.1%)	**.891** ^b^
Race				**.033** ^b^
Asian	41 (2.0%)	12 (3.0%)	53 (2.2%)	
Black or African American	108 (5.3%)	35 (8.8%)	143 (5.9%)	
Hispanic	73 (3.6%)	16 (4.0%)	89 (3.7%)	
Other	413 (20.4%)	67 (16.8%)	480 (19.8%)	
Caucasian or White	1385 (68.6%)	270 (67.5%)	1655 (68.4%)	
Any medical comorbidity	802 (39.7%)	190 (47.5%)	992 (41.0%)	**.004** ^b^
Obesity (body mass index > 30)	596 (29.5%)	133 (33.3%)	729 (30.1%)	**.136** ^b^
ASA score				**.001** ^b^
<3	1793 (88.8%)	332 (83.0%)	2125 (87.8%)	
≥3	227 (11.2%)	68 (17.0%)	295 (12.2%)	
Average mFI-5 Index: mean (SD)	0.039 (0.096)	0.061 (0.115)	0.043 (0.099)	**<.001** ^a^
Diabetes mellitus	84 (4.2%)	21 (5.3%)	105 (4.3%)	**.328** ^b^
Dyspnea symptoms preoperatively	19 (0.9%)	10 (2.5%)	29 (1.2%)	**.009** ^b^
History of severe COPD	14 (0.7%)	8 (2.0%)	22 (0.9%)	**.012** ^b^
Cardiac comorbidity	299 (14.8%)	91 (22.8%)	390 (16.1%)	**<.001** ^b^
Hypertension requiring medication	297 (14.7%)	91 (22.8%)	388 (16.0%)	**<.001** ^b^
Neurological comorbidity	2 (0.1%)	0 (0.0%)	2 (0.1%)	**.529** ^b^
Renal comorbidity	0 (0.0%)	1 (0.3%)	1 (0.0%)	**.025** ^b^
History of bleeding disorders	12 (0.6%)	8 (2.0%)	20 (0.8%)	**.005** ^b^
Hepatic comorbidity	5 (0.2%)	5 (1.3%)	10 (0.4%)	**.004** ^b^
Functional health status prior to surgery				**.002** ^b^
Independent/unknown	2019 (100.0%)	397 (99.3%)	2416 (99.8%)	
Dependent	1 (0.0%)	3 (0.8%)	4 (0.2%)	
Current smoker within 1 year	370 (18.3%)	72 (18.0%)	442 (18.3%)	**.881** ^b^
Steroid use for chronic condition	38 (1.9%)	13 (3.3%)	51 (2.1%)	**.082** ^b^
History of >10% weight loss in 6 months	4 (0.2%)	1 (0.3%)	5 (0.2%)	**.834** ^b^
Preoperative hematocrit <35.5%	39 (90.7%)	19 (100.0%)	58 (93.5%)	.169^b^
Preoperative albumin <3.4 g/dl	5 (1.5%)	5 (4.7%)	10 (2.2%)	**.047** ^b^
Preoperative creatinine >1.21 mg/dl	32 (4.3%)	18 (8.8%)	50 (5.3%)	**.011** ^b^
Surgical setting				**<.001** ^b^
Inpatient	44 (2.2%)	236 (59.0%)	280 (11.6%)	
Outpatient	1976 (97.8%)	164 (41.0%)	2140 (88.4%)	
Principal anesthesia technique				**<.001** ^b^
Epidural	8 (0.4%)	0 (0.0%)	8 (0.3%)	
General	1917 (94.9%)	365 (91.3%)	2282 (94.3%)	
MAC/IV sedation	65 (3.2%)	12 (3.0%)	77 (3.2%)	
Regional	8 (0.4%)	4 (1.0%)	12 (0.5%)	
Spinal	22 (1.1%)	19 (4.8%)	41 (1.7%)	
Quarter of admission				**.048** ^b^
Q1	448 (22.2%)	112 (28.0%)	560 (23.1%)	
Q2	505 (25.0%)	83 (20.8%)	588 (24.3%)	
Q3	515 (25.5%)	94 (23.5%)	609 (25.2%)	
Q4	552 (27.3%)	111 (27.8%)	663 (27.4%)	
Total operation time: mean (SD)	94.4 (51.2)	145.2 (92.6)	102.8 (62.9)	**<.001** ^a^
Total operative time				**<.001** ^b^
≤1.5 h	1088 (53.9%)	128 (32.0%)	1216 (50.2%)	
>1.5 h	932 (46.1%)	272 (68.0%)	1204 (49.8%)	

a Kruskal–Wallis/Wilcoxon signed-rank test. Significant values in bold.

b Chi-squared/Fischer’s exact test.

Unless otherwise specified, history of preoperative comorbidities referred to the period 6 months prior to surgery. Cardiac comorbidities included history of hypertension, congestive heart failure, and prior cardiac surgery. Renal comorbidities included dialysis and history of renal failure. Hepatic comorbidity includes ascites or albumin <3.4 g/dl preoperatively [12]. Available 30-day surgical outcomes included development of any complication, reoperation, readmission, wound complication, VTE, UTI, intraoperative/postoperative bleeding requiring transfusion, and sepsis. Any medical comorbidity variable included obesity, partially and totally dependent functional status, preoperative creatinine >1.21 mg/dl [13], hematocrit <35.5% [14], recent weight loss, and steroid use for a chronic condition. Any complication included reoperation and readmission but did not include longer operative times >1.5 h or LOS >1 days. The median operative times in the A group (87 min) and in the total cohort (90 min) dictated why an operative time >1.5 or <1.5 h was analyzed.

This was a retrospective case-control study, stratified by whether the patient stayed overnight or had ambulatory surgery. Bivariate analysis of prolonged stay was conducted for the entire cohort using the Kruskal–Wallis or Mann–Whitney *U* test for continuous variables and the Chi-squared test or Fischer’s exact test for categorical variables. A similar subanalysis was performed for patients who underwent HA for femoroacetabular impingement (FAI) and/or a labral tear based on CPT codes, as these are highly common indications for HA. Stepwise multivariate logistic regression was used to identify independent predictors of prolonged hospital stay. Variables were entered if *P *< .35 and kept if *P *< .2. All models with more than three levels, such as anesthesia or race, were collapsed into two levels to improve observations and fit of the logistic regression model. Similarly, medical comorbidities with low incidence in the data were removed from selection and represented in the “any medical comorbidity” variable described. The same was done to identify predictors of blood transfusion requirement in a *post hoc* analysis, as this was an unexpected finding in an HA population. Linear regression was used for bivariate analysis of continuous variables like age vs. LOS. All statistics were conducted using SAS software (Cary, NC, USA), wherein *P *< .05 defined statistical significance.

## Results

A total of 2420 patients met inclusion criteria with an average age of 40.0 ± 13.9 years (58.1% female) (Table 2). Of patients, 400 (16.5%) were in the NA group and 2020 (83.4%) were in the A group. Patients were, on average, similar in age, but age stratification showed that patients admitted overnight tended to be 21–30 or >50 years old (*P *= .006). Bivariate analysis showed that Asian and Black/African American patients had higher rates of overnight admission than patients who identified as White/Caucasian or “Other” (*P *= .033). Patients also had significantly higher rates of prolonged stay if they had any medical condition, cardiac comorbidity, hepatic comorbidity, hypertension requiring medication, dyspnea, bleeding disorders, dependent functional status (*P *< .005 for the above variables), COPD, renal comorbidity, preoperative albumin <3.4 g/dl, preoperative creatinine >1.21 mg/dl, or admission in Q1 (*P *< .05 for the remaining variables). No difference in rates of overnight admission was observed among variables of obesity, gender, diabetic history, neurological comorbidity, operative year, steroid use for a chronic condition, or smoking status.

**Figure 1.**
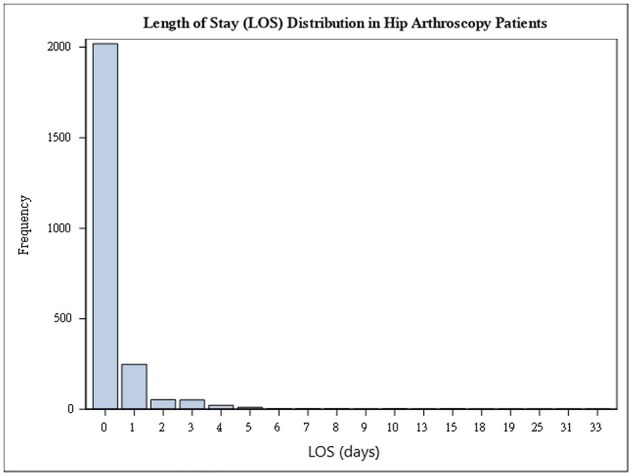
LOS distribution of HA patients shows a significant right skew (Anderson–Darling test: *P *< .005). Median = 0, Q1 = 0, Q3 = 0, Min = 0, and Max = 33.

**Figure 2.**
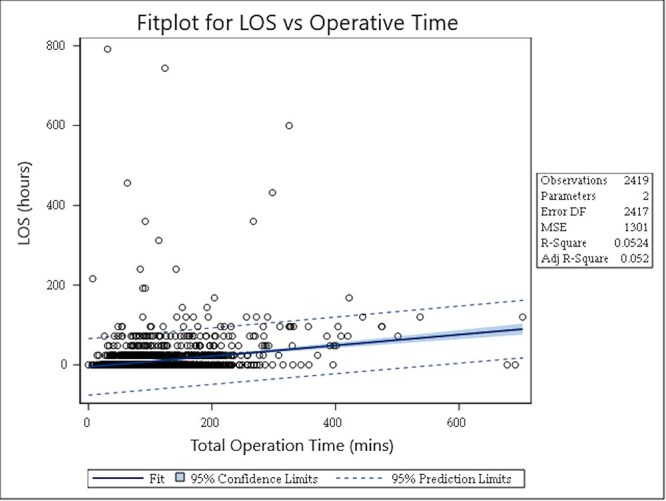
LOS in hours vs. operative time in minutes. Slope = 0.134 ± 0.012, *P* < .001, *R*^2^ = 0.052. For every additional 30 min of operative time, 4.04 ± 0.35 h is added to the LOS.

Regarding surgical outcomes, the NA group had an average LOS of 2.1 days vs. 0 day for the A group. The NA cohort had an average of 145.2 min of operative time compared to 94.4 min in the A cohort (*P *< .001). LOS was right-skewed after exclusion of the previously mentioned outlier patient (Fig. 1). There was a positive correlation between operative time and LOS (slope = 0.134 ± 0.012, *P *< .001, *R*^2^ = 0.052) where for every additional 30 min of operative time, 4.04 ± 0.35 h was added to the LOS (Fig. 2). A higher rate (4.8%) of NA patients used spinal anesthesia compared to 1.1% of A patients, while 1.0% of the NA group used regional anesthesia compared to 0.4% of the A group (*P *< .001). The overall complication rate was 1.9%, with bleeding requiring transfusion being the most common (Table 3). Only 31 patients (1.3%) required a perioperative blood transfusion, and the most significant predictors in this cohort were positive diabetes status, history of bleeding disorder, and higher mFl-5 score >0.2 (Table 4). The mean reoperation rate within 30 days was 0.2%, and the mean readmission rate was 1.2% (NA 3.0% vs. A 0.9%, *P *< .001). The NA group had higher rates of overall complications, readmission, wound complication, sepsis, bleeding complication (*P *< .001 for the above variables), VTE (*P *= .009), and UTI (*P *= .027).

**Table 3.** Perioperative 30-day complications after HA.^a^

	Ambulatory: LOS ≤ 1 day (*N* = 2020)	Nonambulatory: LOS > 1 days (*N* = 401)	Total (*N* = 2420)	*P*-value
Any complication				**<.001** ^b^
No	2012 (99.6%)	362 (90.5%)	2374 (98.1%)	
Yes	8 (0.4%)	38 (9.5%)	46 (1.9%)	
Reoperation				**.648** ^b^
No	2017 (99.9%)	399 (99.8%)	2416 (99.8%)	
Yes	3 (0.1%)	1 (0.3%)	4 (0.2%)	
Readmission				**<.001** ^b^
No	2002 (99.1%)	388 (97.0%)	2390 (98.8%)	
Yes	18 (0.9%)	12 (3.0%)	30 (1.2%)	
Wound complication				**<.001** ^b^
No	2011 (99.6%)	386 (96.5%)	2397 (99.0%)	
Yes	9 (0.4%)	14 (3.5%)	23 (1.0%)	
VTE				**.009** ^b^
No	2018 (99.9%)	397 (99.3%)	2415 (99.8%)	
Yes	2 (0.1%)	3 (0.8%)	5 (0.2%)	
UTI				**.027** ^b^
No	2017 (99.9%)	397 (99.3%)	2414 (99.8%)	
Yes	3 (0.1%)	3 (0.8%)	6 (0.2%)	
Bleeding complications				**<.001** ^b^
No complication	2020 (100.0%)	369 (92.3%)	2389 (98.7%)	
Transfusions intraoperative/postoperative	0 (0.0%)	31 (7.8%)	31 (1.3%)	
Sepsis				**<.001** ^b^
No	2020 (100.0%)	395 (98.8%)	2415 (99.8%)	
Yes	0 (0.0%)	5 (1.3%)	5 (0.2%)	

a All complications could have happened during index hospitalization or at any time in the 30-day postoperative period.

b Chi-squared/Fischer’s exact test.

**Table 4.** Multiple stepwise logistic regression of predictive factors for blood transfusion requirement.

Variable (predictive variable)	OR estimate	OR (95% CI)	
mFI-5 (>0.2 vs. ≤0.2)	33.898	**4.613**	**249.081**
Age group (years)			
21–30 vs. 1–20 (reference)	0.128	**0.039**	**0.420**
31–40 vs. 1–20	0.134	**0.043**	**0.418**
41–50 vs. 1–20	0.087	**0.024**	**0.315**
>50 vs. 1–20	0.139	**0.043**	**0.452**
Female vs. male	2.675	**1.099**	**6.510**
Current smoker within 1 year (yes vs. no)	2.014	0.856	4.738
Diabetes status (no vs. yes)	47.867	**2.659**	**861.705**
Steroid use for chronic condition (yes vs. no)	5.521	**1.341**	**22.731**
Bleeding disorder (yes vs. no)	9.829	**1.620**	**59.638**
General anesthesia (no vs. yes)	5.082	**1.816**	**14.221**

Stepwise logistic regression *c* statistic: .748; Hosmer and Lemeshow test: *P *= .455 and DF = 9. Significant values in bold.

When considering the 1006 patients who underwent HA for FAI and/or a labral tear pathology, bivariate analysis showed that 881 (87.6%) cases were ambulatory with a mean operative time of 102.3 ± 55.8 min. The majority of these patients were 31–40 years old (28.5%), followed by 41–50 years (23.5%) and 21–30 years (23%). Surgeries in Group A were shorter (99.0 ± 52.4 vs. 125.8 ± 71.2 min) and only two (0.2%) procedures experienced a complication compared to three (2.4%) in the NA group (*P *< .001 for both). Similarly, there were only two cases that required an intraoperative or postoperative blood transfusion, and both were in the NA group (*P *< .001). No differences between the groups were identified in the rates of reoperations, readmissions, wound complications, or VTE. A summary of the cohort morbidities for these patients can be found in Table 5.

**Table 5.** HA morbidity bivariate analysis—FAI and labral tear subanalysis.

	Ambulatory: LOS < 1 day (*N* = 881)	Nonambulatory: LOS ≥ 1 days (*N* = 125)	Total (*N* = 1006)	*P*-value
LOS				**<.001^a^**
*N*	880	125	1005	
Mean (SD)	0.0 (0.0)	1.3 (1.0)	0.2 (0.6)	
Median	0.0	1.0	0.0	
Q1, Q3	0.0, 0.0	1.0, 1.0	0.0, 0.0	
Total operation time				**<.001^a^**
*N*	881	125	1006	
Mean (SD)	99.0 (52.4)	125.8 (71.2)	102.3 (55.8)	
Median	91.0	110.0	93.0	
Q1, Q3	65.0, 125.0	71.0, 170.0	65.0, 128.0	
Longop90m				**.002^b^**
≤1.5 h	439 (49.8%)	44 (35.2%)	483 (48.0%)	
>1.5 h	442 (50.2%)	81 (64.8%)	523 (52.0%)	
Principal anesthesia technique				**.134** ^b^
Epidural	1 (0.1%)	0 (0.0%)	1 (0.1%)	
General	859 (97.5%)	122 (97.6%)	981 (97.5%)	
MAC/IV sedation	14 (1.6%)	0 (0.0%)	14 (1.4%)	
Regional	2 (0.2%)	0 (0.0%)	2 (0.2%)	
Spinal	5 (0.6%)	3 (2.4%)	8 (0.8%)	
Any complication				**.001^b^**
No	879 (99.8%)	122 (97.6%)	1001 (99.5%)	
Yes	2 (0.2%)	3 (2.4%)	5 (0.5%)	
Reoperation				**.706** ^b^
No	880 (99.9%)	125 (100.0%)	1005 (99.9%)	
Yes	1 (0.1%)	0 (0.0%)	1 (0.1%)	
Readmission				**.317** ^b^
No	874 (99.2%)	125 (100.0%)	999 (99.3%)	
Yes	7 (0.8%)	0 (0.0%)	7 (0.7%)	
Wound complication				**.607** ^b^
No	877 (99.5%)	124 (99.2%)	1001 (99.5%)	
Yes	4 (0.5%)	1 (0.8%)	5 (0.5%)	
VTE				**.107** ^b^
No	880 (99.9%)	124 (99.2%)	1004 (99.8%)	
Yes	1 (0.1%)	1 (0.8%)	2 (0.2%)	
UTI				^b^
No	881 (100.0%)	125 (100.0%)	1006 (100.0%)	
Bleeding transfusions				**<.001^b^**
No complication	881 (100.0%)	123 (98.4%)	1004 (99.8%)	
Transfusions/intraoperative/postoperative	0 (0.0%)	2 (1.6%)	2 (0.2%)	

a Kruskal–Wallis test.

b Chi-squared test. Significant values in bold.

Stepwise multivariate logistic regression showed that mFI-5 >0 [odds ratio (OR) 1.7, 95% confidence interval (CI) 1.3–2.4], bleeding disorders (OR 3.2, 95% CI 1.3–8.2), and operative time >1.5 h (OR 3.0, 95% CI 2.3–3.8) were independent predictors of prolonged LOS (Table 6). Nongeneral anesthesia (OR 1.9, 95% CI 1.3–2.9) and age groups of 31–40 years (OR 0.4, 95% CI 0.6–0.99) were independent protectors against prolonged stay. Nonambulatory surgery was associated with developing any complication (OR 25.3, 95% CI 11.4–55.7), readmission (OR 2.7, 95% CI 1.3–5.9), and wound complication (OR 7.0, 95% CI 3.0–16.6) as summarized in Table 7. The most common postoperative diagnoses for surgery (Table 8) were labral/cartilage pathology (15.5%), joint ligament sprain (14.9%), and enthesopathy/muscular/fascial pathology (12.2%). Several variables showed significant linear correlation with LOS in hours, including operative time (*P *< .001, *R*^2^ = 0.052, slope = 0.13 ± 0.01 h of LOS/minute of operative time), age [*P *< .001, *R*^2^ = 0.009, slope (*m*) = 0.25 ± 0.05 h of LOS/year of age], mFI-5 (*P *< .001, *R*^2^ = 0.008, *m* = 6.6 ± 1.5 h LOS/0.2 mFI-5), and operative year (*P *= .029, *R*^2^ = 0.002, *m* = 0.88 ± 0.4 h LOS/year).

**Table 6.** Multiple stepwise logistic regression of predictive factors for hospital admission for HA.

Variable	OR (95% CI)	*P*-value
mFI-5 >0 (ref = 0)	1.7 (1.3–2.4)	**.001**
History of bleeding disorders (ref = no comorbidity)	3.2 (1.3–8.2)	**.037**
MAC/regional/sedation/spinal/epidural anesthesia (ref = general anesthesia)	1.9 (1.3–2.9)	**.003**
Operative time >1.5 h (ref ≤ 1.5 h)	3.0 (2.3–3.8)	**<.001**
History of COPD (ref = no comorbidity)	0.5 (0.2–1.2)	.129
ASA ≥ 3 (ref = ASA < 3)	1.4 (0.9–1.99)	.097
Diabetes mellitus (ref = no comorbidity)	0.6 (0.4–1.1)	.128
Age (years) (ref = 18–20 years old)		
21–30	0.9 (0.5–1.4)	.585
31–40	0.6 (0.4–0.99)	**.049**
41–50	0.7 (0.4–1.2)	.157
>50	1.0 (0.6–1.8)	.972
Admission quarter (ref = Q3)		
Q1	1.3 (1.0–1.8)	.065
Q2	0.9 (0.7–1.3)	.590
Q4	1.1 (0.8–1.6)	.399

Stepwise logistic regression *c* statistic: .673; Hosmer and Lemeshow test: *P *= .025 and DF = 8. Significant values in bold.

**Table 7.** Multiple stepwise logistic regression of ambulatory vs. hospital admission for postoperative complications in HA.

Variable (predictive variable)	OR (95% CI)	*P*-value
Any complication^a^		
LOS ≥ 1 (ref = 0)	25.3 (11.4–55.7)	**<.001**
mFI-5 >0 (ref = 0)	0.8 (0.3–2.1)	.657
mFI-5 >0.2 (ref ≥ 0.2)	36.7 (3.4–398.6)	**.003**
Diabetes (ref = none)	19.6 (1.5–248.9)	**.022**
Bleeding disorder (ref = none)	8.3 (2.0–34.4)	**.003**
Age (years) (ref = 18–20 years old)		
21–30	0.1 (0.05–0.5)	**.001**
31–40	0.2 (0.1–0.7)	**.008**
41–50	0.2 (0.07–0.6)	**.005**
>50	0.3 (0.1–0.98)	**.046**
Readmission^b^		
LOS ≥1	2.7 (1.3–5.9)	**.011**
mFI-5 >0	2.1 (0.8–5.1)	.116
Caucasian race (ref = non-Caucasian)	2.5 (1.2–5.3)	**.019**
Steroid use	8.6 (3.1–24.2)	**<.001**
ASA ≥3	2.3 (0.9–6.0)	.084
Reoperation		
LOS ≥1	1.1 (0.1–12.8)	.926
mFI-5 >0	0.01 (<0.01–5.3)	.154
Wound complications^c^		
LOS ≥1	7.0 (3.0–16.6)	**<.001**
mFI-5 >0	1.4 (0.5–4.1)	.500
Dyspnea (ref = no)	10.5 (2.8–39.1)	**<.001**
VTE^d^		
LOS ≥1	6.6 (1.02–42.0)	**.047**
mFI-5 >0	0.3 (0.02–7.2)	.398
Bleeding disorder	21.4 (1.2–378.8)	**.036**
UTI		
LOS ≥1	2.9 (0.5–16.2)	.221
mFI-5 >0	3.5 (0.4–28.0)	.245
Diabetic	28.2 (1.3–661.9)	**.035**
Steroid use for chronic conditions (ref = no steroid)	(18.4–129.1)	**.003**
Bleeding requiring transfusion		
LOS ≥1	999.9 (0.1–999.9)	.932
mFI-5 >0	0.8 (0.3–2.3)	.727
Sepsis		
LOS ≥1	999.9 (0.1–999.9)	.956
mFI-5 >0	0.4 (0.02–7.1)	.505
Dyspnea	102.4 (9.0- >999.9)	**<.001**

Ambulatory vs. hospital admission and mFI-5 were fixed in every logistic regression. Other variables were added. Significant values in bold.

a Variables: age, diabetes, and bleeding disorder. Model *c* statistic: .884; Hosmer and Lemeshow (HL) test: *P *= .818 and DF = 7.

b Variables: White/Caucasian race, steroid use, and ASA. Model *c* statistic: .756; HL: *P *= .401 and DF = 4.

c Variables: dyspnea symptoms. Model *c* statistic: .771; HL: *P *= .578 and DF = 1.

d Variables: chronic steroid use and bleeding disorders. Model *c* statistic: .764; HL: *P *= .609 and DF = 1.

**Table 8.** Postoperative diagnosis frequencies of HA.

Postoperative diagnosis	Ambulatory: LOS < 1 day (*N* = 2020)	Nonambulatory: LOS ≥ 1 days (*N* = 400)	Total	%
Labral/cartilage pathology	320 (15.8%)	54 (13.5%)	374	15.45
Joint ligament sprain*	13 (0.6%)	38 (9.5%)	361	14.92
Enthesopathy/muscular/fascial pathology	263 (13.0%)	32 (8.0%)	295	12.19
Joint pain	246 (12.2%)	38 (9.5%)	284	11.74
Osteoarthritis*	74 (3.7%)	33 (8.3%)	107	4.42
Congenital deformity of hip*	18 (0.9%)	39 (9.8%)	57	2.36
Trochanteric/other bursitis	40 (2.0%)	7 (1.8%)	47	1.94
Synovitis and tenosynovitis	20 (1.0%)	4 (1.00%)	24	0.99
Loose body/fracture	21 (1.0%)	2 (0.5%)	23	0.95
Orthopedic implant complication*	8 (0.4%)	13 (3.3%)	21	0.87
Cyst/neoplasm	14 (0.7%)	5 (1.3%)	19	0.79
Aseptic/osteonecrosis of joint	10 (0.5%)	6 (1.5%)	16	0.66
Joint instability/dislocations	13 (0.6%)	3 (0.8%)	16	0.66
Prosthetic infection/pyogenic arthritis*	0 (0.0%)	12 (3.0%)	12	0.50
Joint contracture	2 (0.1%)	3 (0.8%)	5	0.21
Unspecified derangement of hip	648 (32.1%)	111 (27.8%)	759	31.36
Total	2020 (83.5%)	400 (16.5%)	2421	100.00

Chi-squared test: *P *< .001 and DF = 15.

* *Post hoc* pairwise analysis shows that prosthetic infection/pyogenic arthritis, osteoarthritis, orthopedic implant complication, joint ligament sprain, and congenital deformity of hip had probability values less than the Bonferroni adjusted alpha (.05) of .0015, indicating the statistically significant pairwise difference.

## Discussion

The current study determined that mFI-5 >0, bleeding disorders, operative time >1.5 h, and nongeneral anesthesia were associated with overnight admission after HA. Patients in the NA cohort were also at higher risk of developing any complication, being readmitted, wound complications, and VTE in the first 30 days postoperatively. These findings also suggest that the majority of overnight admissions likely came from the NA group, who tended to have higher mFl-5 scores. Notably, those aged 31–40 years protected against LOS ≥1, while BMI >30, bleeding requiring transfusion, and chronic steroid use produced no difference in rates of overnight admission, as has been identified previously [6]. Interestingly, in the 1006 patients who underwent HA for FAI and/or a labral tear pathology, there were a total of two complications, both in the NA group, and no differences between the groups were identified in the rates of reoperations, readmissions, wound complications, or VTE. This study is unique in its analysis of risk factors for increased hospital stay after HA. While other studies have evaluated risk factors for readmission after HA—namely surgical-site infection, pain, wound complications, and thromboembolic events [2, 6]—they are smaller and do not include specific data on LOS or include a comparative analysis of the resulting risks for later readmission and postoperative complications based on LOS as this study has. Among the orthopedic literature assessing risk factors for increased LOS, most investigations were not performed using an HA population, and therefore they cannot be compared to the findings of this cohort [2, 3, 7].

Hip arthroscopies are generally ambulatory surgeries, and while the overall rate of hospital admission in the current study (16.5%) may be higher than expected for an HA population, none of the FAI/labral tear patients were admitted after surgery, which is typical of these surgeries. Such a difference in admission rates also suggests that many of the total cases in this analysis may have been more involved or performed on patients with greater health complexity than would be found at the typical outpatient surgical center. Still, the overall admission rate was much lower than that reported by Malviya *et al*. [2]. One reason for this could be differences in practice or guidelines from 2005 to 2013 in England, where their surgeries were performed. Another may be that the Hospital Episode Statistics database covers all admissions to English hospital systems, inherently biased to include patients who are likely to be admitted. Third, the codes used to query that database were not listed, and it is possible that the pathologies treated by HA are significantly different and broader than those observed in our cohort, as evidenced by the much larger sample size of ∼6000 patients captured by Malviya *et al*. [2]. Meanwhile, Mei-Dan *et al*. reported that bilateral HA was associated with longer surgical time but not LOS [15]. In that study, all patients, even unilateral cases, were kept overnight and examined before discharge as part of the study protocol. The LOS reported in that study is therefore not comparable to our patients, who are under no such restrictions.

In terms of demographics and comorbidities, prior studies have identified certain risk factors for prolonged hospitalization after surgery, but none have reported an analysis comparable to these findings in the HA population [16–20]. Patients in certain age groups, with Asian or African American backgrounds, and having medical comorbidities were associated with LOS ≥1 in our study. The mFI-5 was also higher in the NA group. Diabetes mellitus, functional status, history of COPD, history of congestive heart failure, and hypertension are all factored in the mFI-5 calculation [11]. With the exception of diabetes and functional status, all mFI-5 variables were also associated with nonambulatory HA. In fact, having any comorbidity in the mFI-5 index increased risk of admission by about two-fold, reinforcing the relevance of this variable to the current study. The quarter of admission and year of surgery had statistically significant but not clinically significant effects on LOS. Even though mFI-5, age, and operative year were positively and statistically significantly correlated with LOS in our study, the low coefficients of determination (*R*^2^) suggest that the low amount of variability in LOS was explained by those predictive variables. This finding also takes into consideration that the average age of our cohort was older than the typical HA patient, further emphasizing that while relevant to patient selection and other surgical outcomes, age is less predictive of increased LOS than some of the other variables identified and was in fact determined to be protective in this analysis.

With regard to operative time, this analysis identified that surgical length >1.5 h was associated with admission after HA. Total operative time showed a significantly positive linear correlation with total LOS where the slope translates to 4.0 h of LOS for every 30 min of operative time, although this may not be clinically significant. Bovonratwet *et al*. also identified longer operative time to be an independent risk factor for overnight admission after HA, albeit in a different patient cohort [21]. They were not able to identify other risk factors for overnight admission. Avoiding prolonged operative time when possible and effectively controlling pain after HA may promote shared decision-making about discharge that avoids unnecessarily prolonging LOS and an increased cost of care.

In addition to decreased surgical time, specific anesthesia and pain management techniques can also help reduce LOS, but no clear consensus exists for HA. Our study found that general anesthesia alone decreased the risk of prolonged stay compared to alternative and adjunctive (monitored anesthesia care, regional, sedation, spinal, or epidural) anesthesia. In contrast, Ward *et al*. previously found that using a femoral nerve block after HA reduced LOS and improved pain relief compared to intravenous morphine [22]. Kunze *et al*. reviewed randomized clinical trials of pain management after HA and determined that two out of seven studies reported that adjunctive anesthesia also reduced LOS, specifically periacetabular local infiltration anesthesia and femoral nerve blocks [23]. On the other hand, four of the remaining seven studies reported no statistical difference in LOS from adjunctive anesthesia: fascial iliac block, lumbar plexus block, extracapsular bupivacaine, and celecoxib. Moreover, Shlaifer *et al*. found that pre-emptive periacetabular bupivacaine analgesia for HA had significantly lower LOS compared to the intra-articular group [24], while Kahlenberg *et al*. found that celecoxib after HA reduced time from operation to discharge, although the total LOS was not reported [25]. While the use of a nerve block in lower extremity surgery has benefits primarily related to postoperative pain control and narcotic use, the variable effects on LOS observed in the literature may be due to increased fall risk, local anesthetic system toxicity, neuropathy, and/or slower return to function [7, 26].

Admission after HA was also associated with increased complications in our study. Previously, the total complication rate after HA reported by Fabricant *et al*. was 1.4–3.8%, with a 0.1% infection rate and even fewer reports of major complications [27]. A later meta-analysis of 6962 hips by Kowalczuk *et al*. demonstrated a similar total complication rate of 4.0% in 2013 [28]. These outcomes correspond well to the rates found in our study (1.9% overall) except for the wound complication rate, which includes infection and dehiscenceand was higher at 1.0%. Among rarer complications, Haskins *et al*. reassuringly reported that intra-abdominal fluid extravasation after HA, a variable not collected in the NSQIP database, did not increase LOS in their 100-patient study [29]. However, multivariate analysis in the current study showed that nonambulatory surgery multiplied the risk of any complication by 25.3, readmission by 2.7, wound complications by 7.0, and VTE by 6.6. Such results demonstrate that the risk of complications produced by admission not only exists, but that it can also be significant. It is therefore fortunate that none of the FAI/labral tear patients required hospital admission, further emphasizing the safety of these procedures. These risks may also be modified by surgeon experience with the procedure(s) performed and could vary depending on the clinical site (e.g. specialized outpatient hip preservation center vs. inpatient). Overall, routine overnight admission after HA should be avoided for a multitude of reasons, not least of all being the increased risks of complication and readmission. However, surgeons should still admit patients when indicated to address any ongoing clinical concerns or immediate surgical complications.

## Limitations

This study shares the same limitations of large database studies. Indications for surgery were not available to be adequately controlled for, including some coding inconsistency in several variables from year to year. The NSQIP database lacks follow-up beyond 30 days to elucidate long-term outcomes like need for revision or total hip arthroplasty. Reasons for overnight admission are also lacking as the NSQIP cannot differentiate between patients admitted overnight due to surgeon preference, surgeon experience, patient preference, or complication. Similarly, NSQIP offers no way to identify if an admission was planned vs. unplanned preoperatively and given the older average age, 1.3% transfusion rate, and higher-than-expected rate of total overnight admissions at 16.5%, there may be a selection bias inherent to use of the database. Another limitation is that admitted patients were fewer than the number discharged, potentially creating a biased comparison of cohorts despite statistical adjustments for non-normal data distributions. Furthermore, the NSQIP does not provide data to quantify the cost effect of prolonged stay, although it is almost invariably greater. These data would be valuable to surgeons in their cost–benefit analysis when considering admission and would be of interest for further study. Finally, the NSQIP captured broad but still limited data from a finite number of hospitals between 2005 and 2017. While outcomes from ambulatory centers and hospitals are captured in this study, the majority come from hospitals and their associated outpatient surgical settings. As such, these findings may not reflect the outcomes or admission thresholds of independent specialty surgical centers that are becoming more commonly used for these types of procedures. This may also be contributory to the observed mean overnight admission rate of 16.5%, as admissions are more likely in the hospital setting due to the availability of resources and medical complexity of patients. Regarding the time range, there have been important evolutions in HA surgical techniques since these data were collected that may affect recent outcomes and, specifically, LOS after these cases. It is likely that if such evolution has had a significant effect, it has been beneficial to outcomes, and this analysis could then have theoretically overestimated rates of postoperative complications.

## Conclusion

HA is a safe procedure with a low overall complication rate. Patients with medical comorbidities captured in higher ASA and mFI-5 scores and those undergoing operations >1.5 h are at greater risk of being admitted to the hospital after HA. Surgeons should optimize controllable risk factors to reduce admissions while ambulatory HA should be performed when possible.

## Data Availability

All data underlying this article were collected from the ACS-NSQIP database with permission via the Participant Use Data File provided by the ACS. The datasets can be made available by request to the ACS at https://www.facs.org/quality-programs/data-and-registries/acs-nsqip/participant-use-data-file/.
